# Well‐Being Therapy as Rehabilitation Therapy for Posttraumatic Stress Disorder Symptoms: A Randomized Controlled Trial

**DOI:** 10.1002/jts.22500

**Published:** 2020-04-14

**Authors:** Mirjam Radstaak, Laura Hüning, Ernst T. Bohlmeijer

**Affiliations:** ^1^ Department of Psychology, Health and Technology University of Twente Enschede The Netherlands; ^2^ Mediant, Community Mental Health Center Enschede The Netherlands

## Abstract

Many individuals with posttraumatic stress disorder (PTSD) continue to have substantial residual symptoms after completing psychological treatment. Well‐being therapy (WBT) has been developed to treat the residual phase of mental disorders, prevent relapse, and promote a full recovery. The present study aimed to compare treatment as usual (TAU) with the long‐term effects of WBT as a rehabilitation therapy in adults who successfully completed psychological treatment for PTSD. Participants who did not meet PTSD diagnostic criteria after completing treatment were randomized to WBT (*n* = 29) or TAU (*n* = 35) groups. Assessments of well‐being, residual PTSD symptoms, and posttraumatic growth were conducted at baseline (T0) and again after 3 months (T1), 6 months (T2), and 1 year (T3). The results of the multilevel analysis revealed that WBT was not more effective than TAU in increasing levels of well‐being, γ = 0.02 (*SE* = 0.11) or posttraumatic growth, γ = 0.10 (*SE* = 0.13) nor in decreasing PTSD symptoms, γ = −0.04 (*SE* = 0.05). However, for participants with low levels of well‐being at baseline (Mental Health Continuum‐Short Form score < 2.6), WBT was more effective than TAU in increasing ratings of well‐being, γ *=* −0.41 (*SE =* 0.19) and posttraumatic growth, γ *=* −0.55 (*SE =* 0.24); this effect was most evident at T3 for posttraumatic growth, *d* = 1.23. Future research should assess clinically relevant individual characteristics that to optimize the effectiveness and utility of WBT.

Posttraumatic stress disorder (PTSD) is a complex and debilitating disorder that has been shown to have a lifetime prevalence of approximately 8% in the general population (Kessler et al., 2005). Many psychological treatments for PTSD have been developed over the past several decades, including exposure therapy, cognitive therapy, cognitive behavioral therapy (CBT), narrative exposure therapy (NET), and eye movement desensitization and reprocessing (EMDR). The results of systematic reviews and meta‐analyses have demonstrated the effectiveness of these treatments across many populations and settings. For instance, various treatments have been shown to be effective for road traffic accident survivors, veterans, survivors of natural disasters, and refugees as well as for individuals traumatized by childhood abuse, sexual abuse, or assaults (e.g., Cusack et al., 2016; Powers, Halpern, Ferenschak, Gilihan, & Foa, 2010).

Although most treatment‐seeking individuals with PTSD respond well to psychological treatment, many continue to have substantial residual symptoms after treatment completion. No specific PTSD symptoms have been shown to be resistant to treatment, and residual symptoms can encompass any PTSD symptom, including unwanted upsetting memories about the trauma, intense negative feelings, or trouble falling asleep (Bradley, Greene, Russ, Dutra, & Western, 2005). In a study of the long‐term effects of cognitive processing therapy and exposure therapy in female rape survivors, Resick et al. (2012) found that for most participants, treatment effects were maintained 5–10 years after therapy. However, 20% of the study participants met the criteria for PTSD during this follow‐up period, which was likely because those participants were unresponsive to the original treatment (Resick et al., 2012). Follow‐up treatments aimed at enhancing and maintaining recovery are therefore important for patients with PTSD who demonstrate suboptimal responses to their original treatment (Pérez Benítez et al., 2012).

Researchers have suggested that assessments of recovery and treatment response should consider not only levels of symptomatology but also levels of well‐being (Fava, Rafanelli, Cazzaro, Conti, & Grandi, 1998): Mental illness and well‐being are related but separate continua (e.g., Keyes, 2005, 2002; Lamers, Westerhof, Bohlmeijer, ten Klooster, & Keyes, 2011). This means that individuals may experience various combinations of high and low levels of mental illness and well‐being, such as high levels of both, low levels of both, or a low level of one coupled with a high level of the other. In one sample, Keyes (2002) found that almost 5% of participants who had been diagnosed as having had experienced a major depressive episode also reported high levels of well‐being. Other research has also demonstrated the importance of well‐being in response to treatment. Impairments in well‐being have been reported in remitted individuals with mood or anxiety disorders (Rafanelli et al., 2000) as well as in those with mild depression (Nierenberg et al, 2010). Moreover, well‐being has been associated with a reduced risk in mental illness with the progression of time (e.g., Keyes, Dhingra, & Simoes, 2010; Lamers, Westerhof, Glas, & Bohlmeijer, 2015; Schotanus‐Dijkstra, ten Have, Lamers, de Graaf, & Bohlmeijer, 2016). When well‐being is incorporated into the definition of recovery, full recovery has not necessarily been achieved even when an intervention has helped an individual to experience a significant decrease in symptom levels. As levels of well‐being could still be low. In one study, researchers found that almost 40% of participants who completed treatment for PTSD still reported low levels of well‐being (Hüning, Radstaak, Lamers, & Bohlmeijer, 2020). It is possible that only interventions that facilitate progress toward restoration or enhancement of well‐being can help a patient reach a full recovery (Fava et al., 1998).

Well‐being therapy (WBT; Fava et al., 1998) was developed to specifically prevent relapse of mental disorders and to promote full recovery. Well‐being therapy is a structured psychological treatment that aims to promote well‐being, corresponding to Ryff's (1989) six dimensions of psychological well‐being: purpose in life, environmental mastery, personal growth, autonomy, self‐acceptance, and positive relationships. The therapy was originally developed to be used as an addition to CBT in treating the residual phase of major depression, with the goal of preventing relapse (Fava et al., 1998). The addition of WBT to CBT was been shown to significantly lower relapse rates among participants with recurring depression at a 6‐year follow‐up compared to clinical management (Fava et al., 2004). Subsequent work extended the approach to other disorders, such as anxiety disorder and cyclothymic disorder, further emphasizing the beneficial effects of WBT (Fava et al., 2005; Fava, Rafanelli, Tomba, Guidi, & Grandi, 2011).

In sum, previous research has revealed that individuals who are treated for PTSD continue to have substantial residual symptoms (Bradley et al., 2005; Resick et al., 2012) and experience low levels of well‐being (Hüning et al., 2020). Low levels of well‐being have been associated with an increased risk of mental illness and relapse (e.g., Schotanus‐Dijkstra et al., 2016; Keyes et al., 2010; Lamers et al., 2015). To prevent relapse and promote full recovery, it is important that PTSD treatments aim to increase well‐being (Fava et al., 2017, 2004; Nierenberg et al, 2010; Rafanelli et al., 2000). The present paper presents the outcomes of a randomized controlled trial that compared the short‐term and follow‐up effects of WBT and treatment‐as‐usual (TAU) in adults with PTSD who did not meet diagnostic criteria for PTSD after treatment completion. We expected that WBT would be more effective than TAU in increasing well‐being and posttraumatic growth (PTG) as well as in reducing PTSD symptoms. Moreover, the current study examined whether differences in well‐being at the start of the treatment would be associated with the effectiveness of WBT.

## Method

### Participants

**Figure 1:**
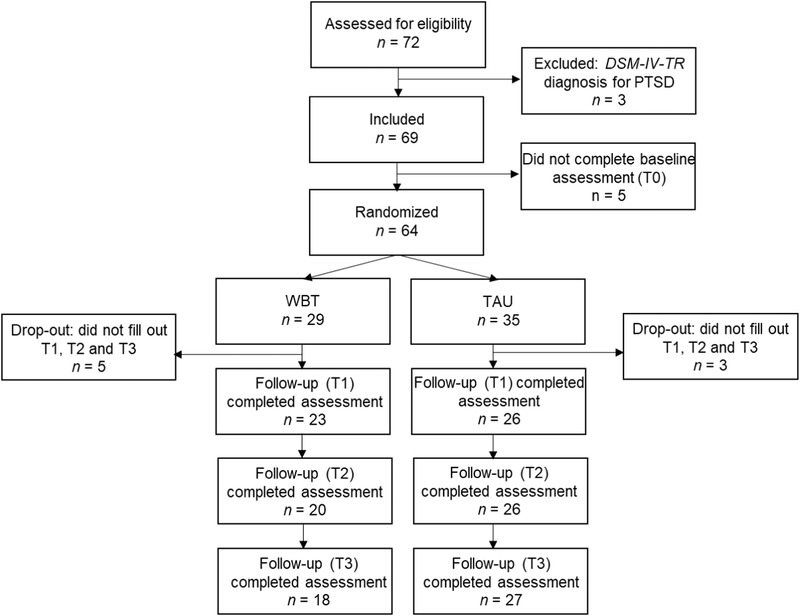
Study profile. *DSM‐IV‐TR* = *Diagnostic and Statistical Manual of Mental Disorders* (4th ed.; text rev.; APA, 2000); WBT = well‐being therapy; PTSD = posttraumatic stress disorder; T1 = Time 1 (3‐month follow‐up); T2 = Time 2 (6‐month follow‐up); T3 = Time 3 (12‐month follow‐up).

A total of 72 individuals were assessed for eligibility and deemed eligible for treatment if they no longer met diagnostic criteria for PTSD after completing treatment for PTSD caused by different types of trauma exposure, such as childhood emotional neglect, sexual abuse, and war trauma. Participants were treated in a Dutch psychotrauma center for outpatient care. Three potential participants were excluded because they still met diagnostic criteria for PTSD per the fourth edition (text revision) of the *Diagnostic and Statistical Manual of Mental Disorders* (*DSM‐IV*‐*TR;* American Psychiatric Association [APA], 2000) after treatment, and five potential participants were eligible but did not complete the baseline assessment (Figure 1). This resulted in the randomization of 64 participants.

An overview of participant characteristics is presented in Table 1. Participants’ age ranged from 18 to 66 years (*M* = 39.83 years, *SD* = 12.92). More than half of the participants were women, and most participants had an intermediate education level and a Dutch cultural background. Most participants were either married (34.4%) or had never been married (40.6%) as opposed to divorced or widowed. There were no significant differences at baseline for any demographic variables or outcome measures between the WBT and TAU groups; thus, the randomization was deemed successful.

**Table 1:** Participant Characteristics

	Total		TAU		WBT	
	(*N* = 64)		(*n* = 35)		(*n* = 29)	
Variable	*M*	*SD*	*M*	*SD*	*M*	*SD*
Age, years^a^	39.83	12.92	38.40	11.58	41.55	14.40
	*n*	%	*n*	%	*n*	%
Female gender^a^	38	59.4	21	60.0	17	58.6
Educational attainment^a^						
Low (primary school, lower vocal education)	10	15.6	5	14.3	5	17.2
Intermediate (secondary school, vocational education)	43	67.2	22	62.9	21	72.4
High (higher vocational education, university)	11	17.2	8	22.9	3	10.3
Marital status^a^						
Never married	26	40.6	14	40.0	12	41.4
Married	22	34.4	12	34.3	10	34.4
Divorced	15	23.4	9	25.7	6	20.7
Widowed	1	1.6	0	0.0	1	3.4
Cultural background^a^						
Dutch	49	76.6	26	74.3	23	79.3
Antillean	1	1.6	0	0.0	1	3.4
Indonesian	2	3.1	1	2.9	1	3.4
Turkish	1	1.6	0	0.0	1	3.4
Mixed	5	7.8	4	11.4	1	3.4
Other	6	9.4	4	11.4	2	6.9

*Note*. TAU = treatment as usual; WBT = well‐being therapy.

a No significant difference between WBT and TAU (*p* < .05).

### Procedure

The study was approved by an independent medical ethics committee for research in the Netherlands (METiGG; NL26248.13.34) and recorded in the Netherlands Trial Register (NTR4424). The study design was a four‐wave randomized controlled trial with two arms: WBT and TAU. Participants filled out questionnaires at baseline (T0) and again after 3 months (T1), 6 months (T2), and 12 months (T3). Almost half the sample (45.7%) had finished treatment at T1, and all participants had finished treatment at T2.

Between March 2014 and July 2016, patients at the psychotrauma center who were at least 18 years of age and had completed treatment for PTSD were approached by their psychologists. All patients had completed one of three treatments: EMDR, prolonged exposure (PE), or NET. Patients were asked whether they wanted to participate in a study that examined the effectiveness of WBT and TAU; those who were interested received a letter with additional information about the research as well as an informed consent form. When individuals gave their informed consent, they were screened for PTSD and other psychological disorders using the Mini International Neuropsychiatric Interview (M.I.N.I.; Sheenan et al., 1998) 1 week after they had completed their treatment. The screening was performed by masters‐level students who were completing an internship; they were trained and supervised by a senior experienced clinical psychologist. Only patients who were successfully treated and no longer met PTSD criteria were included in the study. For this reason, individuals were excluded from participation if they still fulfilled the criteria for a *DSM‐IV*‐*TR* PTSD diagnosis or were in treatment for other psychological disorders. Included participants received a link via e‐mail to fill out the baseline questionnaires. After participants filled out the baseline assessments, they were randomly assigned to one of the two conditions, and the intervention began. Randomization was carried out by research assistants and was stratified according to gender using block randomization. Participants were aware of the rehabilitation therapy they would receive (i.e., WBT or TAU). The link to the follow‐up measurement (T1) was sent 3 months after the participant had filled out the baseline assessments. At T1, 45.7% of participants indicated that they had finished treatment. Six months after participants filled out baseline assessments, they received the link to the next follow‐up measurement (T2). At T2, all participants had completed rehabilitation therapy. One year after participants filled out baseline assessments, they received a link to the last follow‐up measurement (T3). If participants had not filled out the questionnaire within 2 weeks of receiving the link, they were contacted by telephone and reminded to answer the questionnaire. The data collection period ended in August 2017.

#### Well‐being therapy

The goal of WBT was to increase the psychological well‐being of participants by enhancing their levels of purpose in life, environmental mastery, personal growth, autonomy, self‐acceptance, and positive relationships (Ryff, 1989). The therapy was adapted for the Netherlands and consisted of six sessions, with homework assignments, over period of approximately 3 months (Bohlmeijer & Hulsbergen, 2018). All sessions took place at the psychotrauma center, and the same psychologist who guided participants through PTSD treatment also guided them through WBT. All psychologists had a graduate degree in psychology and followed a 2‐year post‐master's training, both of which are required to work in the Dutch healthcare system. Before the therapy started, participants received the self‐help book *Using Positive Psychology Every Day* (Bohlmeijer & Hulsbergen, 2018), with assignments aimed at increasing positive emotions, compassion, and PTG (Schotanus‐Dijkstra, Drossaert, Pieterse, Walburg, & Bohlmeijer, 2015). Before their first session with the psychologist, participants were instructed to recall and write down times in their lives in which they experienced positive emotions. During the first session with the psychologist, these times were discussed, and the importance of positive emotions was explained to the participant. As homework, participants had to continue their positive emotions diary and write down three things that went well for them each day. During the second session, these assignments were discussed, and the importance of compassion and emotion regulation were explained to the client. After this session, participants were instructed to complete assignments every day at home to increase levels of compassion. The third session focused on the importance of cognition in the experience of positive emotions. As homework, participants continued with compassion assignments and were instructed to think of a place where they felt safe and positive (i.e., a place where they could flourish). The fourth and fifth sessions focused on PTG and the importance of coping with traumatic event exposure, and the homework assignments were aimed at increasing PTG. The topic of the sixth session depended on the needs of the individual. Participants were free to choose from different topics, such as self‐development, optimism, and communication. The related homework assignments were given to the participant and discussed during the final session.

#### Treatment as usual

The TAU rehabilitation therapy consisted of three sessions with a psychologist and three sessions conducted via e‐mail. All sessions with the psychologist took place at the psychotrauma center, and every participant was guided by the same psychologist during TAU as they were during PTSD treatment. All psychologists had a graduate degree in psychology and had followed a 2‐year post‐master's training, both of which are required to work in the Dutch healthcare system. During the first TAU session, the psychologist discussed with the participant which area of their life would be the central focus of the therapy, such as work, leisure time, and relationships. Next, the participant and clinician discussed the participant's goals in their chosen area. For the second session, participants were instructed to send an e‐mail to their psychologist stating what they had done to achieve their goals. The psychologist responded to the e‐mail with feedback and gave the participant a new goal‐related assignment. During the third session, participants discussed developments in important life areas, and the psychologist and client discussed the issues on which they would focus their discussions during the next two sessions. Sessions 4 and 5 consisted of e‐mail contact between the client and psychologist, in which the participant reported on the progression of their goals and the psychologist gave feedback and concrete assignments to work on. During the final session, participants were instructed to evaluate their therapy experiences.

#### Treatment integrity

Treatment integrity includes three different aspects: adherence, treatment differentiation, and competence (Waltz, Addis, Koerner, & Jacobsen, 1993). Several actions were taken to ensure treatment integrity. To ensure competence, all psychologists were trained before the start of therapy, and group supervision was conducted throughout the course of the study. Adherence and treatment differentiation—that is, the differences between treatments concerning critical dimensions—were monitored during the study by an independent assessor with a bachelor's degree in psychology. During both WBT and TAU, therapy sessions were videotaped. Two score forms were developed to assess adherence and treatment differentiation. The WBT score form consisted of eight parts: introduction, right assignment during the session, discussion of positive emotions/well‐being, discussion of homework, discussion of the book, doing exercises, bending something negative into something positive, and an invitation to look at the positive side of things. The score form for the TAU consisted of five parts: introduction, ask what is going well, ask what could have been improved, discuss developments in different areas of life, and determine points of attention until the next session. Each part was scored based upon whether it was executed during the therapy session.

#### Adherence

Eleven videotaped therapy sessions (seven WBT sessions and four TAU sessions) were randomly selected and scored. An analysis of the scores revealed psychologist adherence to treatment protocols; WBT had 98.0% strict adherence and TAU had 87.5% strict adherence. To address treatment differentiation, the TAU sessions were also scored on parts of the WBT excluding the introduction. An analysis of the scores revealed psychologists differentiation between treatments. Parts 2–7 of the WBT protocol were scored 0% during TAU. Part 8, an invitation to look at the positive side of things, was scored 100% during TAU because participants were asked what was going well during TAU.

### Measures

#### Well‐being

The primary study measure focused on well‐being. The 14‐item Mental Health Continuum‐Short Form (MHC‐SF; Keyes, 2002) was used to measure three dimensions of well‐being via subscales for Emotional (three items), Social (five items), and Psychological well‐being (six items). Emotional well‐being assessed positive feelings (e.g., “During the past month, how often did you feel happy?”), psychological well‐being assessed feelings about positive functioning in individual life (e.g., “During the past month, how often did you feel that your life has a sense of direction or meaning to it?”), and social well‐being focused on feelings about community life (e.g., “During the past month, how often did you feel that you had something important to contribute to society?”). Participants were required to rate the past‐month frequency of every feeling, using a 6‐point Likert scale that ranged from 0 (*never*) to 5 (*every day*). The mean score for the total scale was calculated, with higher scores indicating higher levels of well‐being. The total MHC‐SF score has demonstrated excellent psychometric properties (Lamers et al., 2011). In the current sample, well‐being was measured at baseline, T1, T2, and T3, and the items showed good reliability, Cronbach's αs = .93–.94.

#### PTSD symptoms

Residual PTSD symptoms were measured using the PTSD Symptom Scale (PSS; Foa, Riggs, Dancu & Rothbaum, 1993) as a secondary outcome measure. The 17‐item PSS measures symptom severity in three dimensions of PTSD: intrusions (five items), avoidance (seven items), and hyperarousal (five items). Participants were asked to rate the past‐week frequency or severity of symptoms, using a 4‐point Likert scale ranging from 0 (*never*) to 3 (*more than 5 times a week*). The mean score of the total scale was calculated, with and higher scores indicating higher levels of PTSD symptoms. The total scale score has demonstrated good test–retest reliability and concurrent validity (Foa et al., 1993). Symptoms of PTSD were measured at all time points, and reliability in the current sample was good, Cronbach's αs = .89–.95.

#### Posttraumatic growth

The Posttraumatic Growth Inventory (PTGI; Tedeschi & Calhoun, 1996) was used as a secondary outcome measure to measure positive outcomes in response to traumatic events. The 21‐item scale measures five dimensions of PTG via subscales for New Possibilities (five items), Relating to Others (seven items), Personal Strength (four items), Spiritual Change (two items), and Appreciation of Life (three items). Participants were asked to rate the answers on a 5‐point Likert scale ranging from 0 (*I did not experience this change as a result of my crisis*) to 4 (*I experienced this change to a very great degree as a result of my crisis*). The scale has demonstrated excellent internal consistency and acceptable test–retest reliability as well as good convergent validity (Tedeschi & Calhoun, 1996). Posttraumatic growth was measured at baseline, T1, T2, and T3. The mean score of the total scale was calculated, with higher scores indicating higher levels of PTG. In the current sample, the reliability of the scale scores was excellent at all time points, Cronbach's α = .96.

#### Depressive symptoms

As PTSD is often comorbid with depression (Flory & Yehuda, 2015), depressive symptoms were measured using the Depression subscale of the Hospital Anxiety and Depression Scale (HADS‐D; Zigmond & Snaith, 1983). This measure uses seven items to measure frequency of depressive symptoms; items are rated on a 4‐point Likert scale. The HADS‐D and has been shown to be a valid and reliable form of measurement (Spinhoven et al., 1997). The mean score of the total scale was calculated, with higher scores indicating higher levels of depressive symptoms. Depressive symptoms were measured at all assessment points, and the scale showed good reliability across all time points, Cronbach's αs = .85–.90.

### Data Analysis

To calculate the necessary sample size, a conservative correlation between the repeated measures was used. An analysis revealed that a sample size of 22 participants was needed to detect a Cohen's *d* effect size 0.40 (Kennard et al., 2014) for the outcome measures. This was based on a four‐wave trial (baseline, T1, T2, T3) with two arms (WBT vs. TAU) using an *r* value of .25 between repeated measures (Guo, Logan, Glueck, & Muller, 2013) and a statistical power of 1–β = 0.95 in a two‐tailed test (*p* < .05). At least 28 participants were needed for randomization when considering a dropout rate of 26.2% during treatment (Fernandez, Salem, Swift, & Ramtahal, 2015).

Statistical analyses were performed with SPSS (Version 24.0). The intention‐to‐treat (ITT) analysis was performed using multilevel analysis. Multilevel analysis is a powerful tool used to analyze data because it allows for random partially missing data to be present without causing complications, and no imputation of data is needed (Singer & Willet, 2003). Missing data varied per measurement moment: At T1, data were available for 49 participants (WBT 20.7% vs. TAU 25.7% missing); at T2, data were available for 46 participants (WBT 31.0% vs. TAU 25.7% missing); and at T3, data were available for 45 participants (WBT 37.9% vs. TAU 22.9% missing). There were no incomplete assessments: All participants filled out the entire test battery at each measurement point. The nonsignificant outcome of the Little's missing completely At random test showed that cases were missing completely at random, χ²(66, *N* = 64) = 77.34, *p* = .160.

To test the study hypotheses, we employed growth curve modeling, a powerful method that can be used to analyze changes over time. The linear change over time was examined, and the values of the time variable represented the differences in time between the measurement moments (values: 1, 1.25, 1.5, and 2.0). Separate analyses were run for well‐being, PTSD symptoms, and PTG. Depressive symptoms were added as a time‐variant covariate to the multilevel analysis, and the interaction between age and time was added as a covariate because age was significantly associated with the study variables (see Supplementary Table S1). The interaction between condition and the linear time trend was added as predictor to the multilevel model to examine whether WBT significantly increased well‐being and PTG as well as whether it significantly decreased PTSD symptoms compared to TAU. Next, we examined whether differences in well‐being at the start of the treatment were associated with the effectiveness of WBT. Researchers have suggested that a score below 2.6 on the MHC‐SF requires treatment (Franken, de Vos, Westerhof & Bohlmeijer, 2019). Therefore, two groups were created: a group of participants with low ratings of well‐being (score less than 2.6 at baseline) and a group of participants with higher ratings of well‐being (score of 2.6 or higher at baseline). The interaction between well‐being statuses (lower vs. higher) as well as the interaction between well‐being status (lower vs. higher), condition, and time were added as predictors to the multilevel model to examine whether levels of well‐being at the start of the treatment was associated with changes over time in the study variables and whether well‐being at the start of the treatment interacted with treatment outcomes. Analysis of covariances (ANCOVA) were used to examine significant interactions.

The improvement of model fit was tested using the decrease in ‐2 log‐likelihood compared to the model that included the intercept and the slope. The heterogenous first‐order autoregressive covariance structure was used, as variances were assumed to be heterogenous due to the different time intervals between measurements. Restricted maximum likelihood (REML) estimation was used to estimate the parameters of the multilevel model. Effect sizes at T1, T2, and T3 were calculated with Cohen's *d* (Cohen, 1992). Cohen's *d* was calculated for the differences between the conditions (TAU and WBT) for participants with lower levels of well‐being and higher levels of well‐being at baseline. The confidence intervals were calculated to examine whether effect sizes differed significantly from zero.

## Results

Table 2 displays the descriptive statistics for the study. The outcomes of the multilevel analysis are presented in Table 3. The fit statistics improved for all dependent variables compared to the unconditional growth model. The significant intercepts show the average levels at which participants experienced well‐being, PTSD symptoms, and PTG at baseline. The significant effect of depressive symptoms showed that at each assessment point throughout the study, higher levels of depression predicted lower levels of well‐being and PTG as well as higher levels of PTSD symptoms. The significant main effect of time for well‐being and PTSD symptoms indicated that there was a linear change in levels of well‐being and PTSD symptoms. Older age was associated with a linear increase in PTSD symptoms and a linear decrease in PTG. The interaction between condition (WBT vs. TAU) and time was not significant. These outcomes did not support our expectations as WBT was not associated with linear increases in well‐being or PTG nor was it associated with linear decreases in PTSD symptoms during the study.

**Table 2:** Overview of Means and Standard Deviations for Well‐Being (WB), Posttraumatic Stress Disorder (PTSD) Symptoms, and Posttraumatic Growth (PTG) at Baseline and Follow‐Up Assessments

	TAU									WBT								
	Lower WB			Higher WB			Total			Lower WB			Higher WB			Total		
Assessment Point	*n*	*M*	*SD*	*n*	*M*	*SD*	*n*	*M*	*SD*	*n*	*M*	*SD*	*n*	*M*	*SD*	*n*	*M*	*SD*
WB^a^ (range: 0–5)																		
T0	17	1.78	0.57	18	3.73	0.63	35	2.79	1.15	11	2.19	0.38	18	3.47	0.45	29	2.99	0.76
T1	11	2.38	0.93	15	3.81	0.76	26	3.20	1.09	10	2.73	0.71	13	3.50	0.84	23	3.16	0.87
T2	12	2.51	0.57	14	3.70	0.62	26	3.15	0.84	8	2.63	0.96	12	3.63	0.88	20	3.23	1.02
T3	12	2.41	0.88	15	3.71	0.82	27	3.14	1.06	6	3.04	0.67	12	3.49	0.87	18	3.34	0.82
PTSD (range: 0–3)																		
T0	17	0.87	0.48	18	0.37	0.26	35	0.61	0.46	11	0.90	0.46	18	0.37	0.24	29	0.57	0.43
T1	11	0.91	0.73	15	0.36	0.42	26	0.60	0.62	10	0.75	0.53	13	0.40	0.31	23	0.55	0.45
T2	12	0.87	0.61	14	0.52	0.45	26	0.68	0.55	8	0.74	0.49	12	0.37	0.40	20	0.52	0.46
T3	12	0.92	0.74	15	0.55	0.61	27	0.71	0.68	6	0.87	0.68	12	0.43	0.51	18	0.58	0.59
PTG (range: 0–5)																		
T0	17	2.08	1.04	18	3.32	0.97	35	2.72	1.18	11	2.52	1.28	14	3.11	0.98	29	2.89	1.12
T1	11	2.25	1.08	15	3.51	0.99	26	2.98	1.20	10	3.12	1.13	13	3.23	1.16	23	3.18	1.13
T2	12	2.25	0.71	14	3.21	0.95	26	2.77	0.96	8	2.73	1.58	12	3.25	1.17	20	3.04	1.34
T3	12	2.25	0.87	15	3.31	1.29	27	2.84	1.22	6	3.33	0.89	12	3.28	0.91	18	3.30	0.88

*Note*. TAU = treatment as usual; WBT = well‐being therapy; T0 = Time 0 (baseline); T1 = Time 1 (T2; 3‐month follow‐up); T2 = Time 2 (6‐month follow‐up); T3 = Time 3 (12‐month follow‐up).

a Norm scores for well‐being: Healthy sample: *M =* 3.98 (*SD* = 0.85; Lamers et al., 2011); sample with pathology: *M* = 1.90 (*SD* = 1.00; Franken, Lamers, Ten Klooster, Bohlmeijer, Westerhof, 2018). No mean norm scores for other assessments were available.

**Table 3:** Multilevel Analyses With Well‐Being (WB), Posttraumatic Stress Disorder (PTSD) Symptoms, and Posttraumatic Growth (PTG) as Dependent Variables

	WB				PTSD				PTG			
Variable	γ	SE γ	γ	SE γ	γ	SE γ	γ	SE γ	γ	SE γ	γ	SE γ
Intercept	3.32***	0.15	3.28***	0.16	0.15	0.08	0.17*	0.08	3.34***	0.20	3.33***	0.20
Time‐variant covariate												
Depressive symptoms	−0.93***	0.09	−0.83***	0.09	0.52***	0.05	0.49*	0.05	−1.00***	0.11	−0.98***	0.11
Rate of change												
Time	0.22*	0.10	−0.17	0.14	0.12*	0.06	0.19*	0.07	0.09	0.12	−0.09	0.14
Age × Time	−0.01	0.00	−0.01*	0.00	0.01*	0.00	0.01*	0.00	−0.01*	0.00	−0.01**	0.00
Condition^a^ × Time	0.02	0.11	0.24	0.14	−0.04	0.05	−0.08	0.08	0.10	0.13	0.43*	0.19
Well‐Being at T0^2^ ×Time			0.69***	0.12			−0.13	0.07			0.30	0.15
Condition^a^ × Well‐Being at T0^b^ × Time^c^			−0.41*	0.19			0.07	0.11			−0.55*	0.24
Δ‐2 log‐likelihood	−77.32***, *df* = 3		−15.97***, *df* = 2		−.76.98***, *df* = 3		3.08, *df* = 2		−65.39***, *df* = 3		−1.96, *df* = 2	

*Note*. ^a^Treatment as usual = 0, well‐being therapy = 1. ^b^Well‐being at T0: lower well‐being = 0; higher well‐being = 1. ^c^The interaction between well‐being, condition, and time was not significant when well‐being was added as a continuous variable was to the multilevel model.

^*^ *p* < .05. ^**^ *p* < .01. ^***^ *p* < .001.

Adding well‐being (lower vs. higher) to the multilevel model improved model fit for well‐being only. The interaction between well‐being and time was significant for well‐being: Participants with low levels of well‐being at baseline showed higher increases in well‐being than participants with higher levels of well‐being at baseline. The interaction between well‐being (lower vs. higher), condition (WBT vs. TAU), and time was significant for well‐being and PTG. However, the ANCOVAs with the between‐subjects factors of condition (WBT vs. TAU) and well‐being (lower vs. higher) as well as covariate levels of well‐being or PTG at baseline did not reveal any significant effects for well‐being at T1, *F*(1, 48) = 0.04, *p* = .851; T2, *F*(1, 38) = 0.03, *p* = .854; or T3, *F*(1, 44) = 0.27, *p* = .603; or for PTG at T1, *F*(1, 48) = 0.13, *p* = .720; T2, *F*(1, 38) = 0.19, *p* = .668; or T3, *F*(1, 43) = 1.28, *p* = .265.

The effect sizes for the different assessment points are shown in Table 4. The effect sizes suggest that participants with low levels of well‐being benefitted more from WBT, whereas participants with higher levels of well‐being benefitted more from TAU. This was especially apparent at T3, as participants with low levels of well‐being benefitted more from WBT than TAU, which was reflected in a large and significant effect size for PTG.

**Table 4:** Effect Sizes for the Differences Between Treatment as Usual (TAU) and Well‐Being Therapy (WBT) at Time 1, Time 2, and Time 3, and for Lower and Higher Levels of Well‐Being

	TAU vs. WBT^a^					
	Time 1		Time 2		Time 3	
Condition and WB rating	*d*	95% CI	*d*	95% CI	*d*	95% CI
WB						
Lower WB	0.41	[−0.46, 1.27]	0.17	[−0.73,1.07]	0.77	[−0.26, 1.77]
Higher WB	−0.38	[−0.37, 1.13]	−0.11	[−0.67, 0.88]	−0.27	[−0.50, 1.03]
Total	0.04	[−0.52 – 0.60]	0.08	[−0.50, 0.66]	0.21	[−0.39, 0.81]
PTSD						
Lower WB	0.25	[−0.61, 1.11]	0.23	[−0.67, 1.12]	0.07	[−0.92, 1.04]
Higher WB	−0.10	[−0.64, 0.84]	0.35	[−0.43, 1.12]	0.20	[−0.56, 0.96]
Total	0.08	[−0.48, 0.64]	0.32	[−0.27, 0.90]	0.20	[−0.40, 0.80]
PTG						
Lower WB	0.79	[−0.11, 1.67]	0.43	[−0.49, 1.32]	1.23*	[0.14, 2.28]
Higher WB	−0.25	[−0.49, 1.00]	0.04	[−0.74, 0.81]	−0.02	[−0.74, 0.78]
Total	0.18	[−0.39, 0.74]	0.24	[−0.35, 0.82]	0.41	[−0.20, 1.02]

*Note*. WB = well‐being; PTSD = posttraumatic stress disorder symptoms; PTG = posttraumatic growth.

a A negative effect size indicates that TAU was more effective than WBT.

^*^ *p* < .05.

## Discussion

The present study examined the short‐term and follow‐up effects of WBT compared to TAU with regards to promoting well‐being and full recovery in patients who had previously completed treatment for PTSD. The results revealed that WBT was not more effective than TAU in increasing well‐being or PTG or for decreasing PTSD symptoms. Although this finding contradicts the results of earlier studies, which showed that WBT decreased psychological symptoms (Fava et al., 1998, 2005, 2011), we attribute our findings to differences in inclusion and exclusion criteria. In previous studies, participants with a *DSM* PTSD diagnosis were included (Fava et al., 1998, 2005, 2011), whereas a PTSD diagnosis was an exclusion criterion in our study. Therefore, levels of PTSD symptoms at the start of WBT were relatively low, which may explain the nonsignificant effects.

The present findings suggest that future researchers may wish to examine the individual characteristics that optimize the effectiveness of WBT. More specifically, future research could examine whether low levels of well‐being at the start of the treatment (i.e., a score lower than 2.6 on the MHC‐SF) are associated with the effectiveness of WBT. The cutoff score of 2.6 was based on the assumption that a minimally clinically important difference is reflected in changes of one‐half of a standard deviation in outcome measures (Sloan, Symonds, Vargas‐Chanes, & Fridley, 2003;), and, therefore, a score that is one‐half of a standard deviation below the mean of the general population was suggested as a way to distinguish between low and normal functioning (Franken et al., 2019; Lamers et al., 2011). The difference in response to WBT or TAU underlines the urge to develop evidence‐based methods for individualizing treatments. Personalized interventions for mental health should include reliable assessments of clinically relevant individual characteristics, such as differences in well‐being before the start of treatment. This would help to tailor treatments to those individual characteristics and optimize treatment effectiveness (Ng & Weisz, 2016).

At T3, which was 1 year after the start of rehabilitation therapy, our results revealed the highest effect sizes that favored WBT over TAU for participants with low levels of well‐being at the start of treatment. Although the effect size for well‐being was not significant, the effect sizes for well‐being and PTG at T3 were high compared to the long‐term effects of psychotherapy for PTSD (Kline, Cooper, Rytwinski, & Feeny, 2018). To our knowledge, only two studies have examined the effect of WBT on levels of well‐being, and the results were mixed. One study found positive effects of WBT on well‐being directly after treatment (Fava et al., 2005), whereas the other study found no significant effects (Fava et al., 1998). Neither study examined the long‐term effects of WBT on well‐being. Well‐being and mental illness are related but separate continua (e.g., Keyes, 2002, 2005; Lamers et al., 2011); therefore, it may be possible that the short‐term effects of WBT shown in a previous study (Fava et al., 2005) were caused by reductions in symptoms, whereas the long‐term effects of WBT are caused by increases in well‐being. Bringing a person out of a state of negative functioning may take less time than facilitating progression toward restoration of the positive, such as having a purpose in life, feelings of personal growth, and building positive relationships (Ryff & Singer, 1996).

The results of our study give preliminary evidence that WBT might help individuals with low levels of well‐being cope with trauma and facilitate growth (i.e., PTG) in response to traumatic events; WBT might benefit PTG by increasing personal and environmental resources that are needed for PTG, such as feelings of mastery and belonging (Woodward & Joseph, 2003). It is unlikely that distorted positive illusions, such as self‐aggrandizement or an exaggerated sense of personal control and unrealistic optimism, which have been associated with PTG, influenced the perception of PTG in our study (Maercker & Zoellner, 2004). All participants in in the current study sought and completed treatment for PTSD, which makes it unlikely that they suffered from an exaggerated sense of personal control and unrealistic optimism. Moreover, the effects on PTG emerged at least 6 months after WBT. Positive illusions are more likely to emerge directly after a traumatic event, whereas adaptive PTG increases once a longer period of time has elapsed after the traumatic event (Helgeson, Reynold & Tomich, 2006; Marcker & Zoellner, 2004).

There are several limitations that need to be considered when interpreting the current findings. First, our sample size was relatively small. The power analysis showed that our sample had enough power to examine the effectiveness of WBT compared to TAU. Nonetheless, the power might have been insufficient to examine the interaction between WBT and TAU for participants with lower versus higher levels of well‐being at the start of the therapy. The number of participants with low levels of well‐being was small, and this might explain why the multilevel analysis did reveal a significant interaction effect even though the post hoc tests were overall not significant. Second, another major limitation was that the follow‐up measurements were not scheduled after therapy was finished but rather at time points 3, 6, and 12 months after the start of WBT or TAU. Therefore, not every participant had completed the rehabilitation therapy by T1. This is a possible explanation as to why effect sizes were small at this measurement point and why it took longer for the effects of WBT to show. Third, we distinguished participants with lower or higher well‐being based on a cutoff score of 2.6 on the MHC‐SF (Franken et al., 2019), but no other cutoff scores were examined. Regression to the mean (RTM) could also have influenced the changes in well‐being between the lower and higher well‐being groups. This occurs when unusually large measurements are used to divide groups and when there is a weak correlation between the variable used to divide the groups and outcome measure (Barnett, Van der Pols, & Dobson, 2004). In the present study, no unusually large measurements were used to divide groups, and the variable used to divide groups was related to the outcome measures; therefore, the effects of RTM on the outcomes is likely to be negligible. Fourth, the psychologists were aware of the possible beneficial effects of WBT, and this might have influenced the outcomes of the study. However, it is unlikely that the expectations of the psychologists favored WBT, as the positive effects of WBT emerged at least 6 months after the treatment was completed. Fifth, we used an active control condition, which was one of the strengths of the current study, but there were more face‐to‐face meetings with the psychologist during WBT than during TAU. Although nonspecific treatment factors, such as attention, are recognized to be important for therapeutic effects, they are unlikely to explain long‐term effects (Li et al., 2015). Sixth, the determination that PTSD was no longer present was based on *DSM‐IV*‐*TR* criteria and not the current criteria from the fifth edition of the *DSM* (*DSM‐5*). In a national sample of 2,953 U.S. adults, prevalence rates of PTSD were slightly lower when *DSM‐5* criteria for PTSD was utilized compared to *DSM‐IV*‐*TR* criteria; however, the biggest difference in the prevalence rate was reported as 1.6% (Kilpatrick et al., 2013). This small difference in prevalence is unlikely to have affected the inclusion of our relatively small group of participants. Finally, the study was conducted in a Dutch psychotrauma center for outpatient care and, therefore, may not be generalizable to other treatment settings or patient populations in other countries. The strengths of this study included the randomized allocation of treatment, the 1‐year longitudinal design, the active control group, and the training and supervision of all psychologists. This study was also unique as it was the first, to our knowledge, to examine WBT as rehabilitation therapy for PTSD.

Future research should examine whether WBT is effective for participants with a current *DSM* PTSD diagnosis. There are several reasons why this might be interesting. First, past research has shown that WBT decreases symptoms of psychopathology for participants who still meet *DSM* criteria (Fava et al., 1998, 2005, 2011). Second, a considerable number of patients with PTSD do not seem to respond to traditional treatments (Resick et al., 2012), and new evidence‐based treatments are needed for this hard‐to‐treat group. Third, the preliminary results of our study suggest that WBT could be more effective for individuals with PTSD who report low levels of well‐being, and individuals with severe PTSD symptoms might also experience these feelings of suboptimal well‐being (Keyes, 2005). Future research might wish to examine the individual characteristics that influence treatment outcomes of WBT to tailor and optimize treatments to those characteristics (Ng & Weisz, 2016). For instance, WBT might be especially effective for patients with PTSD and comorbid depressive symptoms, as WBT was initially developed for patients with depression (Fava et al., 1998). Other factors that may also increase positive outcomes of WBT are an appreciation of life (Hagenaars & van Minnen, 2010) or being part of a minority group (Helgeson et al., 2006).

To conclude, the present study examined the effects of WBT as compared to TAU to promote well‐being and full recovery in patients who successfully completed treatment for PTSD. The results revealed that WBT was not more effective than TAU in increasing well‐being or PTG nor was it more effective in decreasing PTSD symptoms during the study. Although the findings of our study are preliminary and inconclusive, results suggest that participants who reported low levels of well‐being at the start of the treatment benefitted more from WBT than TAU. Future research should assess clinically relevant individual characteristics to optimize the effectiveness and utility of WBT.
